# Effects of auditory and visual stimuli on glucose metabolism in Holstein dairy cattle

**DOI:** 10.1186/s13028-018-0436-y

**Published:** 2019-01-05

**Authors:** Leslie Antonio González-Grajales, Laura Pieper, Philipp Görner, Stefan Görner, Rudolf Staufenbiel

**Affiliations:** 1 Ruminant and Swine Clinic, Department of Veterinary Medicine, Free University of Berlin, Koenigsweg 67, 14163 Berlin, Germany; 2 Institute for Veterinary Epidemiology and Biostatistics, Department of Veterinary Medicine, Free University of Berlin, Koenigsweg 65, 14163 Berlin, Germany

**Keywords:** Cattle, Glucose tolerance test, Insulin, Noise

## Abstract

**Background:**

Standardization of the intravenous glucose tolerance test (ivGTT) in cattle has received little attention despite its widespread use to monitor glucose metabolism. The impact of management practices including several sensorial stimuli on test responses has not yet been described in young cattle. The objective of this study was to analyze the effects of noise exposure, and visual food stimuli in combination with physical restraint on ivGTT and insulin traits in Holstein cattle. A total of 108 ivGTT (6 tests per animal) were performed in bulls (n = 6), steers (n = 6), and heifers (n = 6) aged 312 to 344 days. The main parameters analyzed for glucose and insulin included: basal concentration (G0, Ins0), maximum concentration (GMAX, InsMAX), and final concentration at 63 min (G63, Ins63), glucose and insulin area under the curve (GAUC, InsAUC), and glucose half-life time (GHLT). Noise stimuli were induced by playing rock music at approximately 90 dB either before (NI) or immediately after glucose injection (NII). Visual food stimuli were induced by feeding the neighboring animals while the tested animal was restrained in a headlock.

**Results:**

Almost all glucose and insulin traits were affected by gender (P< 0.05) whereas the factor with least impact on ivGTT was NI. InsMAX and InsAUC were affected (P < 0.002) by all factors analyzed. GHLT and G63 were affected by gender and noise with higher values in bulls when compared to steers and heifers. Furthermore, InsAUC and InsMAX values derived from NII significantly differed in bulls when compared to steers and heifers. Significantly higher values for G0 (P < 0.001), InsMAX (P < 0.001) and InsAUC (P = 0.001) were observed when exposed to the visual food stimulus whereas GMAX (P = 0.02) and GAUC (P = 0.04) decreased. Higher Ins63 values were observed in bulls exposed to the visual food stimulus when compared to heifers.

**Conclusions:**

Short-term exposure to noise and visual food stimuli might lead to variations in glucose metabolism and insulin secretion which emphasizes the necessity to avoid practices involving auditory or visual stimuli prior to or during the conduction of an ivGTT.

## Background

Frequent monitoring of management practices is essential to identify factors generating stress in dairy and beef cattle farms [1]. Sounds, commonly defined as noise, might trigger stress responses attributed to painful stimuli or altered behavior affecting several organs [2]. For instance, the level of noise in livestock production, measured in the frequencies from 25 to 35 kHz (i.e. audible range for cattle [3]) has been associated not only with increased respiratory and heart rate [4] but also with altered production traits [5]. Therefore, avoiding noise-derived stressful situations seem to lead to better welfare [6]. Nevertheless, there is little evidence about the reference values of unwanted sounds for most management systems although it is known that cattle are able to respond with changes in the neuroendocrine system when stressors are intermittent [7]. Additionally, evidence suggests that carbohydrate metabolism is affected in ruminants exposed to a range of noises produced by an industrial engine or human vocalizations [2]. However, alterations in glucose tolerance and insulin concentrations as a result of noise exposure in dairy or beef cattle have not yet been reported.

One of the most common methods to study glucose metabolism in cattle includes the calculation of glucose removal rates from the blood stream under the influence of insulin commonly performed using an intravenous glucose tolerance test (ivGTT) [8, 9]. Changes on glucose concentration at specific time points post glucose infusion reflect the ability of an organism to rapidly utilize the metabolite which comprises the individual glucose and insulin reaction [10]. The information obtained from these responses is routinely used to assess conditions such as insulin resistance [11], diagnose diabetes, although its incidence in cattle is very low [12], determine the impact of certain compounds on insulin sensitivity [13] among other applications. Despite the wide implementation of ivGTT in cattle, careful data interpretation is always recommended because test standardization is lacking [14]. Recently, the effects of food deprivation times [14] and glucose dose [15] on ivGTT and insulin traits have been demonstrated. Similarly, the consequences of some management practices involving auditory and visual stimuli taking place during conduction of ivGTT might alter the ability of the different organs to use and dispose glucose and insulin; however, reports on this field are unavailable in cattle. Current animal housing facilities are surrounded by a wide range of auditory stimuli derived mostly from human activities which represent not only a potential source of animal discomfort but also a confounding factor when assessing glucose and insulin concentrations [16]. Thus, we aimed to determine the influence of short-term auditory and visual food stimuli on glucose metabolism and insulin concentration in young Holstein cattle.

## Methods

### Animals

Eighteen animals were purchased from a commercial dairy farm located in Brandenburg, Germany. Holstein bulls (n = 6), steers (n = 6), and heifers (n = 6) were included in the study. Castration was performed 10 wk prior to study conduction. Experiments took place after a period of 8 wk used for acclimation to the new diet, housing, and personnel. At the beginning of the study, bulls, steers, and heifers were 318 (SD = 16.3), 312 (SD = 46.3), and 344 days (SD = 16.2) old, respectively. Body weights (BW) were different among groups: bulls, steers, and heifers had BW of 364 (SD = 21.8), 335 (SD = 22.1), and 347 kg (SD = 32.2), respectively. Animals were housed in individual stalls with headlocks on straw bedding in experimental facilities at the Ruminant and Swine Clinic, Free University of Berlin, Germany.

### Study design

Animals were enrolled during two seasons: (1) May to November and (2) December to May of the following year. Different individuals were used during the two periods. Each individual was tested 6 times namely on day 1, 8, 10, 15, 22, and 36. A total of 108 ivGTT were carried out. Tests 1, 4 and 6 served as controls. Results from these tests derived from undisturbed animals (no auditory or visual stimuli were implemented). During tests 2 and 3, noises were induced and for test 5 a visual food stimulus was applied.

### Diet

Animals were fed a diet containing 1 kg hay and 1.5 kg concentrate offered four times a day, and straw ad libitum. Feed analysis and ration composition are shown in Table 1.

**Table 1:** Feed analyses and ration composition of experimental diet

	Hay	Pellet concentrate	Wheat straw	Calculated ration composition
DM in OM (%)	86	89	87	88
Ash (g/kg) in DM	109	61	81	80
Metabolizable energy (MJ/kg) in DM	9.4	12.1	6.2	10.9
Crude protein (g/kg) in DM	138	140	32	135
Crude fiber (g/kg) in DM	293	80	489	176
Crude fat (g/kg) in DM	32	36	12	34
Starch (g/kg) in DM	0	260	11	152
Sugar (g/kg) in DM	ND	82	ND	48
Calcium (g/kg) in DM	9.4	6.1	3.1	8.3
Phosphorus (g/kg) in DM	3.1	2.9	0.8	3.3
Sodium (g/kg) in DM	0.8	2.4	1.3	1.7
Magnesium (g/kg) in DM	1.9	2.3	1.0	2.1
*kg OM* ^a^	1^a^	1.5^a^	0.1^a^	–

*DM* dry matter, *OM* organic matter, *ND* not determined

^a^Ration given 4 times/day

### Acoustic exposure

To determine the influence of noise on glucose tolerance, animals were exposed to loud rock music, noises provoked by beating a metal bar against a metal shovel combined by human vocalizations close to the animal’s head at approximately 90 dB. At test number 2, the noise was induced immediately prior to conduction of ivGTT (glucose injection) and was defined as noise I (NI). The same noise exposure was repeated at the following examination (test number 3) but it was initiated once glucose injection was completed and ended before the second blood collection at min 7. This experiment was defined as noise II (NII). Both noise exposures lasted for 5 min.

### Visual food stimuli

At test number 5, animals were subjected to visual food stimuli under physical restraint by fixing the head in the headlock and feeding behavior from neighboring animals prior to the test. Glucose infusion started immediately after visual stimuli. For that, fed animals were located directly next to the food-restricted group on both sides of the stall. Grain (1.5 kg) was offered to fed animals and ivGTT was conducted only on food-restricted animals. This experiment examined the influence of short-term food restriction and visual food stimulus on glucose tolerance test traits. Each visual exposure stimulus lasted until the non-deprived animals finished their ration (~ 5–10 min). Animal test allocation was reversed on the following day. Animals fed on the previous day were now food-restricted and subjected to ivGTT.

### Glucose tolerance test

Animals were fasted for 12 h prior to ivGTT with water available at all the times. On testing days, they were only loosely fixed on the neck using a head halter attached to headlocks. Subsequently, an indwelling 14 G × 8 cc cannula (Melsungen AG, Melsungen, Germany) was inserted in the jugular vein and fixed to the skin to minimize the effects of handling on glucose metabolism. Animals were accustomed to the personal and very often lying behavior during blood collection was observed. The ivGTT was conducted as described previously [17]. Briefly, the first blood sample was obtained immediately prior to glucose i.v. administration using a 40% solution (B Braun Vet Care, Melsungen AG, Melsungen, Germany) at 1 g/kg BW^0.75^. Glucose administration lasted for 0.5 to 1 min followed by catheter flushing with 0.9% NaCl solution (Isotone Kochsalz-Lösung, B Braun Melsungen AG, Melsungen, Germany). The blood samples were drawn every 7 min until min 63 for a total of 10 individual samples. Serum was recovered and stored at − 18 °C until analyzed.

Serum glucose concentration was estimated by the hexokinase method using the Gluco-quant Glucose/HK Cobas^®^ test (Roche Diagnostics GmbH, Mannheim, Germany) according to the manufacturer’s instructions using an automatic spectrophotometer analyzer (Cobas Mira Plus CC^®^, Roche Diagnostic GmbH, Mannheim, Germany). Minimal detectable concentration for this method was 0.11 mmol/L and intra-and inter-assay coefficient of variation were 1.8 and 4.4%, respectively. Insulin concentration was assessed by solid phase radioimmunoassay using a commercial kit (Radioimmuno-coat-A-count insulin^®^, DPC Bierman GmbH, Germany) as described by Kremer [18]. Analytical sensitivity was 1.2 µIU/mL and intra-and inter-assay coefficient of variation were 5.0 to 6.3% and 5.6 to 12.4%, respectively.

Parameters analyzed for ivGTT included: basal glucose (G0) and insulin concentration (Ins0), maximum glucose (GMAX) and insulin concentration (InsMAX), glucose (G63) and insulin (Ins63) concentration at the end of the test, glucose half-life time (GHLT), and area under the curve for glucose (GAUC) and insulin (InsAUC). GHLT was calculated as described by Kaneko et al. [8] using linear regression analysis of the natural logarithm of the glucose concentrations as follows:

$$ {\text{GHLT}}\, = \,\left[ {{{{ \ln }\left( 2\right)} \mathord{\left/ {\vphantom {{{ \ln }\left( 2\right)} k}} \right. \kern-0pt} k}} \right]\,*\, 100 \, \left( { \hbox{min} } \right) $$

The letter (*k*) represents a coefficient determined by the following formula:

$$ k\, = \,\left[ {{ \ln }\left( {\text{G14}} \right)\, - \,{ \ln }\left( {\text{G42}} \right)} \right]/\left( {{\text{T42}}\, - \,{\text{T14}}} \right)\,*\, 100 \, \left( {\% /{ \hbox{min} }} \right) $$

From the previous formula, letters G and T represent glucose concentrations and time points in minutes post glucose infusion, respectively. Numbers 14 and 42 denote min post glucose injection. Area under the curve was estimated using the trapezoidal rule.

### Statistical analyses

**Fig. 1:**
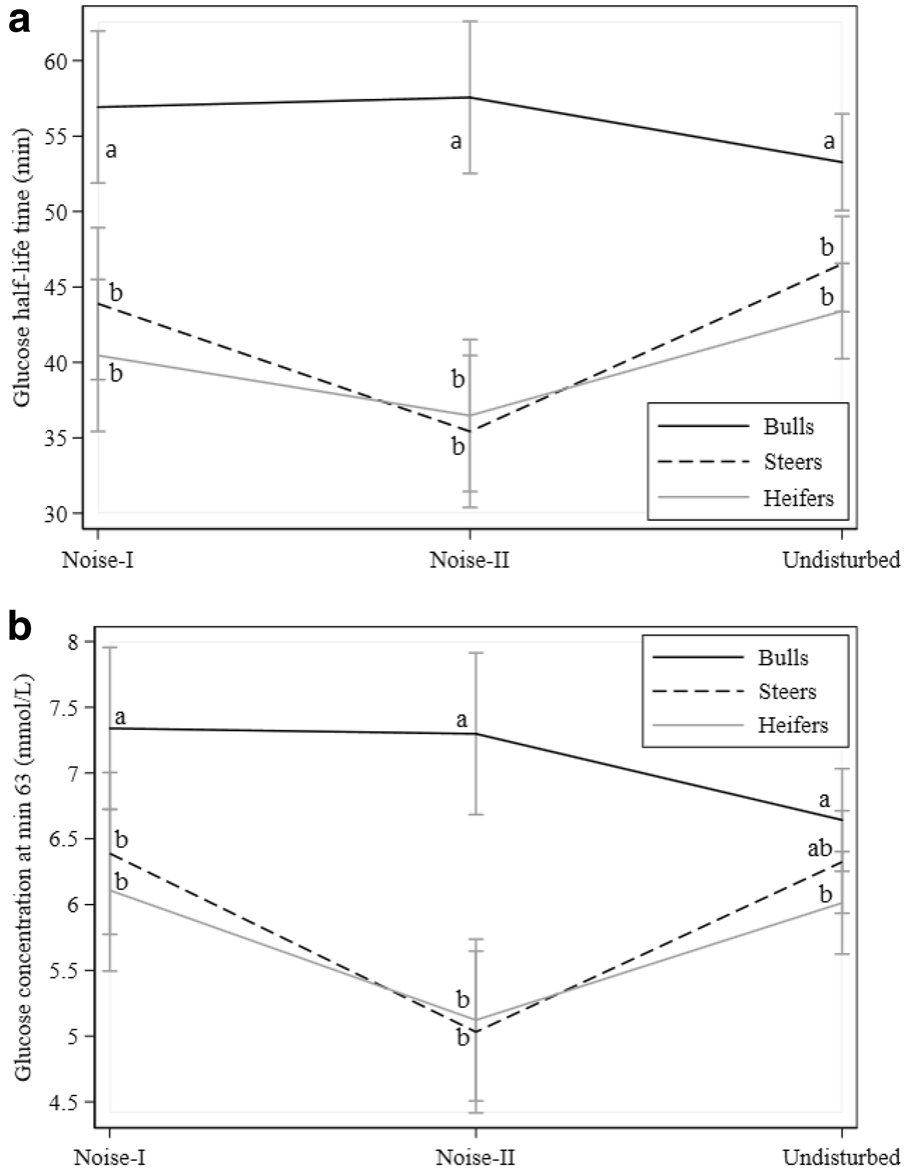
Effects of noise on glucose traits: **a** glucose half-life time, and **b** glucose concentration at min 63. Black, dotted, and gray lines denote bulls, steers, and heifers, respectively. Different letters indicate statistically significant differences between groups

**Fig. 2:**
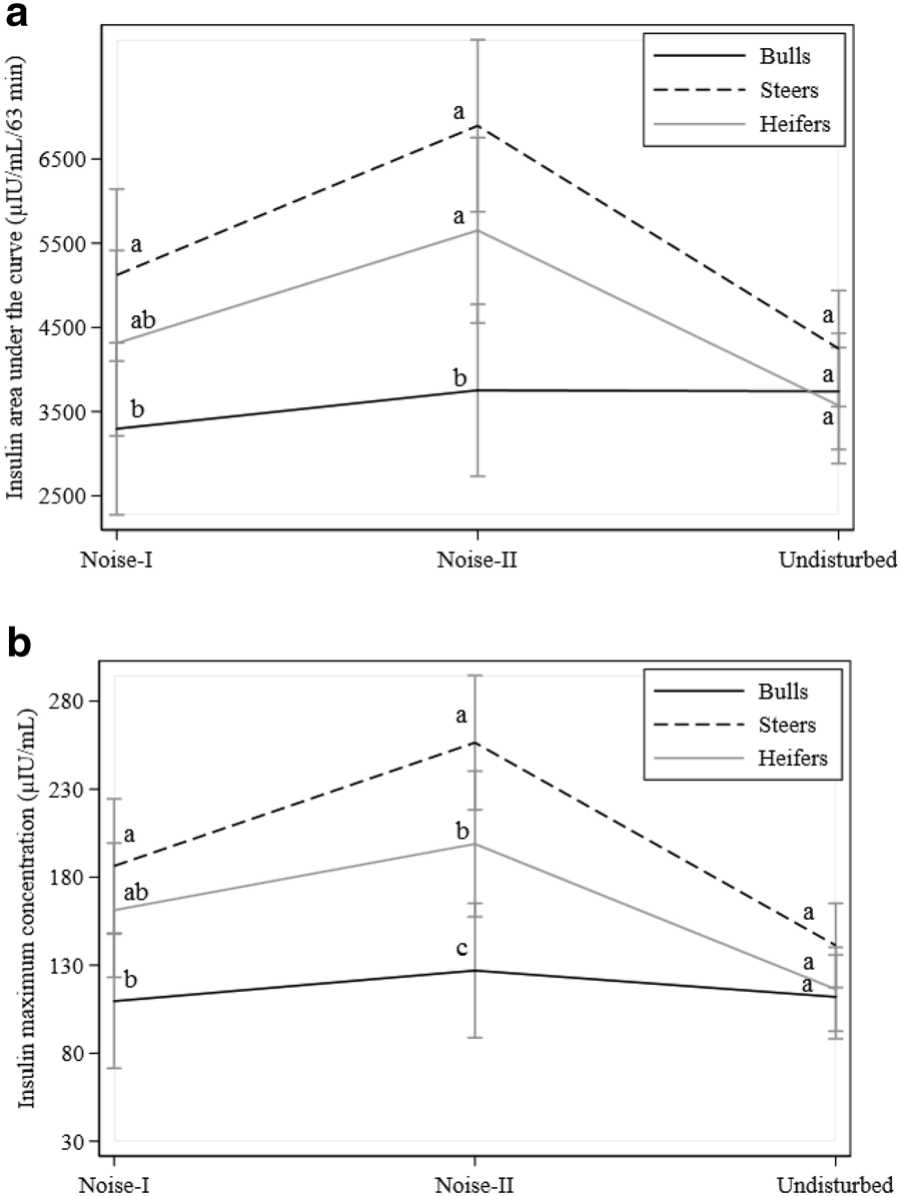
Effects of noise on insulin traits: **a** insulin area under the curve, and **b** insulin maximum concentration. Black, dotted, and gray lines denote bulls, steers, and heifers, respectively. Different letters indicate statistically significant differences between groups

**Fig. 3:**
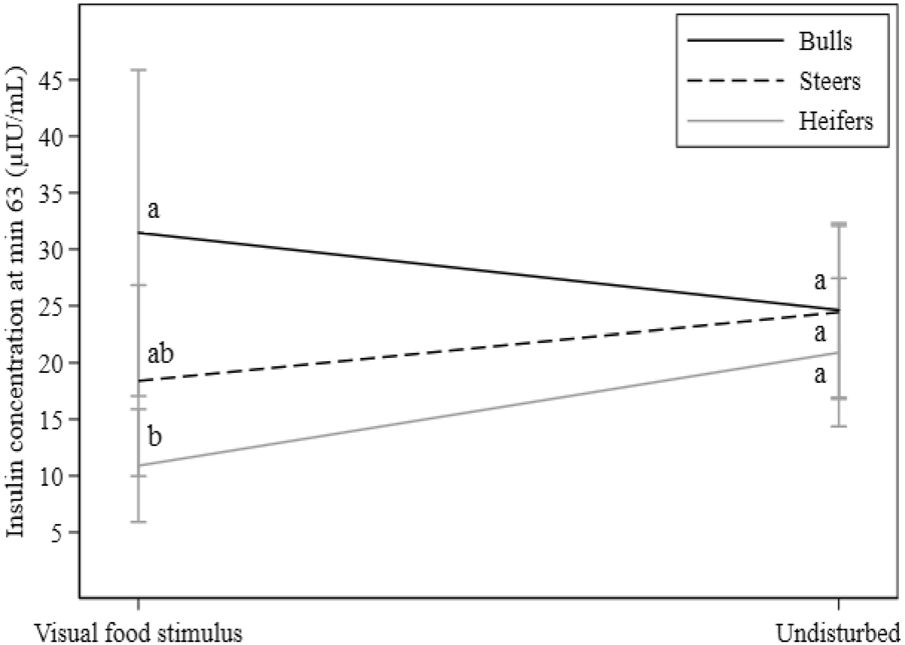
Effects of deprivation food on insulin concentration at min 63. Black, dotted, and gray lines denote bulls, steers, and heifers, respectively. Different letters indicate statistically significant differences between groups. Values were back-transformed from the log scale for representation

Glucose and insulin traits derived from each examination were analyzed for normal distribution using graphical methods and Shapiro–Wilk test. All glucose and two insulin traits (InsMAX, InsAUC) were normally distributed, whereas base-10 logarithmic transformation was applied to Ins0 and Ins63. Results were back-transformed for graphical representation. Descriptive statistics included median, percentiles 25 and 75, maximum and minimum values (Table 2). Total number of observations was 108 for three glucose and two insulin traits, whereas 1–3 observations were removed from the calculations for each of the remaining glucose and insulin variables due to extreme deviations (more than 2.5 ± SD compared to the group mean). Sample size is shown in Tables 3 and 4. Generalized linear mixed models were implemented to determine the influence of gender, noise, and visual food stimulus on glucose and insulin. Interactions between food stimulus and noise with gender were tested in the model and significant interactions were presented (Figs. 1, 2, 3). Animal was included as a random effect in all models to account for repeated measurements in the same tested animals. Residuals were analyzed for outliers, homoscedasticity, and normal distribution. Statistical analyses were carried out with SPSS^®^ version 22 (IBM Deutschland GmbH, Ehningen). Statistical significance was set at P < 0.05.

**Table 2:** Descriptive statistics for intravenous glucose tolerance test and insulin traits

Variable	n	Median	Percentile 25	Percentile 75	Minimum	Maximum
G0 (mmol/L)	108	4.99	4.76	5.37	3.90	5.84
GMAX (mmol/L)	108	13.8	13.3	14.4	12.1	16.1
G63 (mmol/L)	108	6.26	5.56	6.99	3.52	8.06
GHLT (min)	108	46.7	40.4	54.4	26.6	67.3
GAUC (mmol/L/63 min)	108	204.6	178.9	226.5	129.7	269.7
Ins0 (μIU/mL)	108	8.82	6.79	11.5	4.39	28.9
InsMAX (μIU/mL)	108	128.4	93.0	176.4	51.7	326.2
Ins63 (μIU/mL)	108	22.5	15.1	33.5	6.5	102.1
InsAUC (μIU/mL/63 min)	108	3977.0	2983.7	5213.4	1741.2	9968.1

*G0* basal glucose concentration, *GMAX* maximum increase in glucose concentration, *G63* glucose concentration at min 63, *GHLT* glucose half-life time, *GAUC* glucose area under the curve, *Ins0* basal insulin concentration prior to glucose injection, *InsMax* maximum increase in blood insulin concentration, *Ins63* insulin concentration at min 63, *InsAUC* insulin area under the curve

**Table 3:** Generalized linear mixed-models for gender, and management factors on i.v. glucose tolerance test traits

Fixed effects	G0^a^ mmol/L			GMAX mmol/L			G63 mmol/L			GHLT^b^ min			GAUC mmol/L/63 min		
	ß	SE	P	ß	SE	P	ß	SE	P	ß	SE	P	ß	SE	P
Intercept	4.70	0.08	< 0.001	14.2	0.15	< 0.001	5.97	0.22	< 0.001	44.02	1.56	< 0.001	204.27	4.09	< 0.001
Gender			< 0.001			0.03			< 0.001			< 0.001			*ns*
Bull	0.57	0.11	< 0.001	− 0.52	0.21	0.01	0.64	0.31	0.04	10.17	2.23	< 0.001	–	–	–
Steer	0.21	0.11	0.05	− 0.40	0.21	0.05	0.30	0.31	0.34	3.30	2.21	0.13	–	–	–
Heifer	ref	ref	ref	ref	ref	ref	ref	ref	ref	ref	ref	ref	ref	ref	ref
Noise (N)			*ns*			*ns*			< 0.001			< 0.001			*ns*
I	–	–	–	–	–	–	0.12	0.28	0.66	− 3.51	2.50	0.16	–	–	–
II	–	–	–	–	–	–	− 0.89	0.26	0.001	− 7.49	2.23	0.001	–	–	–
Undisturbed	–	–	–	–	–	–	ref	ref	ref	ref	ref	ref	–	–	–
Food stimuli (FS)			< 0.001			0.02			*ns*			*ns*			0.04
Food-stimulated	0.19	0.05	< 0.001	− 0.36	0.16	0.02	–	–	–	–	–	–	− 14.12	7.09	0.04
Undisturbed	ref	ref	ref	ref	ref	ref	–	–	–	–	–	–	ref	ref	ref
Gender*N			*ns*			*ns*			0.001			< 0.001			*ns*
[Bull]*[N I]	–	–	–	–	–	–	0.40	0.40	0.31	5.87	3.55	0.10	–	–	–
[Bull]*[N II]	–	–	–	–	–	–	1.31	0.37	0.001	10.79	3.18	0.001	–	–	–
[Bull]*[undisturbed]	–	–	–	–	–	–	ref	ref	ref	ref	ref	ref	–	–	–
[Steer]*[N I]	–	–	–	–	–	–	− 0.07	0.40	0.86	− 0.09	3.54	0.98	–	–	–
[Steer]*[N II]	–	–	–	–	–	–	− 0.29	0.37	0.43	− 4.15	3.16	0.19	–	–	–
[Steer]*[undisturbed]	–	–	–	–	–	–	ref	ref	ref	ref	ref	ref			

*ß* coefficient, *G0* basal glucose concentration prior to injection, *GMAX* maximum increase in blood glucose concentration, *G63* glucose concentrations at min 63, *GHLT* glucose half-life time, *GAUC* glucose area under the curve, *N* noise, *FS* food stimuli, *ns* no statistically significant difference, *–* not shown because values were not statistical significant, *ref* referent category

^a^G0 was estimated based on n = 105

^b^GHLTwas estimated based on n = 107

**Table 4:** Generalized linear mixed-model for gender, and management factors on insulin traits

Fixed effects	Ins0 µIU/mL			InsMAX^a^ µIU/mL			Ins63 µIU/mL			InsAUC^b^ µIU/ml/63 min		
	ß	SE	P	ß	SE	P	ß	SE	P	ß	SE	P
Intercept	0.34	0.03	< 0.001	105.85	12.57	< 0.001	1.31	0.07	< 0.001	3313.4	353.6	< 0.001
Gender			*ns*			< 0.001			0.03			0.002
Bull	–	–	–	− 1.05	17.23	0.95	0.09	0.10	0.36	225.4	487.7	0.64
Steer	–	–	–	30.01	17.23	0.08	0.04	0.10	0.68	731.3	487.7	0.13
Heifer	–	–	–	ref	ref	ref	ref	ref	ref	ref	ref	ref
Noise (N)			*ns*			< 0.001			*ns*			< 0.001
I	–	–	–	47.13	20.80	0.02	–	–	–	851.5	622.9	0.17
II	–	–	–	81.71	18.30	< 0.001	–	–	–	2126.6	441.5	< 0.001
Undisturbed	–	–	–	ref	ref	ref	–	–	–	ref	ref	ref
Food stimuli (FS)			*ns*			< 0.001			0.02			0.001
Food-stimulated	–	–	–	50.49	11.43	< 0.001	− 0.25	0.06	< 0.001	993.9	281.1	0.001
Undisturbed	–	–	–	ref	ref	ref	ref	ref	ref	ref	ref	ref
Gender*N			*ns*			0.001			*ns*			< 0.001
[Bull]*[N I]	–	–	–	− 53.87	29.10	0.06	–	–	–	− 1290.1	839.1	0.12
[Bull]*[N II]	–	–	–	− 67.29	24.57	0.007	–	–	–	− 2047.3	591.3	0.001
[Bull]*[Undisturbed]	–	–	–	ref	ref	ref	–	–	–	ref	ref	ref
[Steer]*[N I]	–	–	–	− 9.63	29.10	0.74	–	–	–	− 7.16	839,1	0.99
[Steer]*[N II]	–	–	–	34.36	24.57	0.16	–	–	–	594.4	591.3	0.31
[Steer]*[Undisturbed]	–	–	–	ref	ref	ref	–	–	–	ref	ref	ref
Gender*FS			*ns*			*ns*			< 0.001			*ns*
[Bull]*[Food-restricted]	–	–	–	–	–	–	0.35	0.08	< 0.001	–	–	–
[Bull]*[Undisturbed]	–	–	–	–	–	–	ref	ref	ref	–	–	–
[Steer]*[Food-restricted]	–	–	–	–	–	–	0.19	0.08	0.02	–	–	–
[Steer]*[Undisturbed]	–	–	–	–	–	–	ref	ref	ref	–	–	–

Values from Ins0 and Ins63 derived from base-10 logarithmic transformations

*ref* reference values, *ß* coefficient, *Ins0* basal insulin concentration prior to glucose injection, *InsMAX* maximum increase in blood insulin concentration, *Ins63* insulin concentrations at 63 min, *InsAUC* insulin area under the curve, *N* noise, *FS* food stimuli, *ns* no statistically significant difference, *–* not shown because values were not statistically significant, *ref* referent category

^a^InsMax was estimated based on n = 107

^b^InsAUC was estimated based on n = 106

## Results

### Descriptive statistics

Descriptive statistics for glucose and insulin are displayed in Table 2. On average, G0 was 4.99 mmol/L [inter quartile range (ICR) 4.76–5.37] and increased 8.8 mmol/L to a GMAX of 13.8 mmol/L (ICR 13.3–14.4). At min 63, glucose concentration was 6.26 mmol/L (ICR 5.56–6.99) and GHLT 46.7 min (ICR 40.4–54.4). Variability in insulin values was much greater in all measurements. The median Ins0 was at 8.82 μIU/mL (ICR 6.79–11.5) and increased by 119.6 μIU/mL to an InsMax of 128.4 (ICR 93.0–176.4 μIU/mL). At the last measurement, Ins63 decreased to 22.5 μIU/mL (ICR 15.1–33.5).

### Gender

Gender had an influence on most of the parameters assessed (except Ins0 and GAUC; Tables 3 and 4). Due to the interaction effects, coefficients for this independent variable cannot be interpreted without taking these interactions into account. Nevertheless, values for steers were often between those of heifers and bulls but not significantly different from heifers (Tables 3 and 4). On the other hand, bulls had significantly different values from those of heifers for most parameters.

### Noise

Noise had an effect on GHLT, G63, InsAUC, and InsMAX (P < 0.05, Tables 3 and 4) and interaction of noise with gender was present for these traits (Figs. 1 and 2). Compared to undisturbed animals, GHLT values from heifers and steers decreased with NII exposure while values from bulls increased (Fig. 1a). Similarly, G63 values decreased in heifers and steers with NII and increased in bulls (Fig. 1b). However, highest values for G63 in bulls were observed when exposed to NI. For InsAUC and InsMAX (Fig. 2a, b), values for heifers and steers increased with NII and returned to close to undisturbed values for NI exposure. On the other hand, values in bulls almost remained unchanged for InsAUC and InsMAX.

### Visual food stimulus

Visual food stimuli and feeding behavior from neighboring animals influenced most glucose and insulin traits in tested animals (Tables 3 and 4, Fig. 3). Food-stimulated animals showed increased values for G0 (0.19 mmol/L, P < 0.001), InsMAX (50.5 μIU/mL, P < 0.001), and InsAUC (993.9 μIU/mL/63 min, P = 0.001) when compared to control animals (undisturbed). Significant differences were also found between food-stimulated and control animals for GMAX (− 0.36 mmol/L, P = 0.02) and GAUC (− 14.12 mmol/L/63 min, P = 0.04) with decreasing values in the former group. Ins63 increased when exposed to the visual food stimulus in bulls but decreased in heifers (Table 4, Fig. 3) when compared to control animals; however, no effect was observed in steers. The food stimulus did not affect G63, GHLT, and Ins0.

## Discussion

The impact of unwanted sounds commonly defined as “noises” on health has received considerable attention in animal and human studies [19, 20]. However, studies focusing on the effects of noise on glucose tolerance during short-time exposure in cattle are limited. Consequently, it is imperative to determine if noises derived from animal husbandries or humans could alter the responses obtained from an ivGTT. In humans, it has been shown that noise exposure (45 dB) during four consecutive nights altered glucose metabolism in healthy participants under controlled conditions [21]. This study reported significantly decreased basal glucose concentrations and elevated glucose and insulin area under the curve in the exposed group. Engine noises around 97 dB were associated with increased blood glucose concentrations and leucocyte counts in dairy cows [2]. However, neither glucose elimination nor insulin secretion rates were reported. Furthermore, rodents exposed to long-term noises developed insulin resistance and increased stress hormones concentrations [22]. The investigators introduced noises at 95 dB to male mice during 4 h for 1 day and reported decreased glucose tolerance characterized by significantly higher GMAX and GAUC when compared to the control group. Daily noise exposure for 10 or 20 days resulted in significantly higher insulin (InsMAX, InsAUC). Interestingly, these last traits were also affected in our experiments despite the animals were only exposed for 5 min prior to ivGTT (NI) or during the first 5 min after glucose injection (NII). ivGTT traits from animals exposed to NI showed no differences when compared to undisturbed animals but findings derived from NII evidenced significantly lower G63, GHLT, and higher insulin values (e.g. InsMAX, InsAUC) resulting in increased glucose tolerance. The differences in G63, GHLT were only observed in heifers and steers which might be explained by their increased sensitivity to sudden environmental stimuli (sound and motion) as reported by Lanier et al. [23]. Other experiments have found gender differences on behavior and cortisol levels in testosterone propionate-treated heifers exposed to fear [21]. For instance, an association between steroid administration and fearfulness reduction to novel objects or unfamiliar surroundings due to the inhibitory functions of testosterone on adrenal function has been documented [24]. Thus, differences in GHLT and G63 traits in animals exposed to noise might be attributed to steroid concentrations with statistically significant higher values in bulls when compared to steers and heifers. One limitation of the present study is that stress hormones during and after the ivGTT were not assessed. Consequently, underlying the mechanisms that could explain differences in glucose and insulin traits due to alterations on the hypothalamus–pituitary–adrenal axis (HPAA) remain elusive. However, evidence showed that steers at 15–16 months old had statistically significant elevated cortisol concentrations prior to truck loading when compared to bulls [25]. This difference might be attributed to impaired feedback at the HPAA in castrated animals [26, 27] affecting glucose tolerance and insulin concentration as observed in our study. Nevertheless, strong relationships between serum glucose and cortisol concentrations have been reported [28].

Cephalic phase insulin release (CPIR, i.e. the insulin secretion by the pancreas during the cephalic phase of digestion) occurs prior to or during food ingestion and it is caused by neuro-mediated sensory stimuli such as sight, smell or taste [29, 30]. Visual food stimuli experiments conducted in monogastric species reported rapid elevation in basal insulin concentration due to increased epinephrine release [31]. In lactating cows, food presentation for few min prior to its consumption resulted in significant increases in glucose and insulin concentrations [32] whereas other reports have only found similar responses within 2–10 min after feeding in cows [33] and steers [34]. Interestingly, ewes which underwent vagotomy showed a significant increase in plasma insulin concentrations 2 min after the presentation of food with elevated values for 6 min [35]. Similarly, in our study almost all ivGTT traits for glucose and insulin were affected by the visual food stimulus except for G63, GHLT, and Ins0. Therefore, CPIR might account for the elevated insulin concentrations in InsAUC and InsMAX found in this study. However, because assessments of insulin concentration during the first min after food exposure were not performed, we can only speculate a primary role of food-related sensory stimuli on rapid insulin release. On the other hand, one limitation of the current study is that the visual food stimulus was also accompanied by physical restraint which could cause anxiety in the experimental animals with subsequent activation of the hypothalamic–pituitary–adrenal axis. This experiment was intended to combine both stimuli (physical restraint and visual food stimulus) because during the conduction of an ivGTT, animal fixation is commonly used to obtain periodic blood samples. Despite the CPIR has been reported in ruminants [35], physical restraint and individual frustration due to inability to access food might also explain our findings. A combination of factors including early insulin and stress-induced epinephrine secretion, and steroidal hormone concentration might clarify the responses observed in glucose tolerance. A study conducted by Lewis [36] found increased general activity, frustration behavior and significantly elevated cortisol concentrations in Cotswold pigs when they were unable to access food stored in lidded feeders. Thus, it is possible that food presentation stimulated neural control of insulin secretion as observed in other species [29]. Our results evidenced the importance of avoiding feeding practices and auditory stimuli around the time of ivGTT implementation to minimize confounding factors. Standardized test conditions and calm handling have to be ensured to obtain reliable data. Furthermore, there were significant gender differences regarding the reaction to the stimuli applied in this study. Steers were metabolically more similar to heifers than to bulls. This indicates that heifers and steers might be more prone to stressful disturbances involving noises whereas bulls might be more sensitive to frustrating situations, such as observing fed stall mates.

## Conclusions

Glucose tolerance test and insulin traits were highly affected by noises and visual food stimuli. These effects have to be considered and avoided when conducting the test for research or diagnostic purposes.

## Abbreviations

G0: basal glucose concentration
Ins0: basal insulin concentration
BW: body weight
CPIR: cephalic phase insulin release
FS: food stimuli
GAUC: glucose area under the curve
G63: glucose concentration at min 63
GHLT: glucose half-life time
HPAA: hypothalamus pituitary adrenal axis
InsAUC: insulin area under the curve
Ins63: insulin concentration at min 63
ICR: inter quartile range
ivGTT: intravenous glucose tolerance test
GMAX: maximum glucose concentration
InsMAX: maximum insulin concentration
NII: noise stimulus after glucose infusion
NI: noise stimulus prior to glucose infusion
